# A 14-day limit for bioethics: the debate over human embryo research

**DOI:** 10.1186/s12910-017-0198-5

**Published:** 2017-05-30

**Authors:** Giulia Cavaliere

**Affiliations:** Department of Global Health & Social Medicine, King’s College London, London, UK

**Keywords:** Embryo research, Value pluralism, Compromise, Beneficence, Warnock report

## Abstract

**Background:**

This article explores the reasons in favour of revising and extending the current 14-day statutory limit to maintaining human embryos in culture. This limit is enshrined in law in over a dozen countries, including the United Kingdom. In two recently published studies (2016), scientists have shown that embryos can be sustained in vitro for about 13 days after fertilisation. Positive reactions to these results have gone hand in hand with calls for revising the 14-day rule, which only allows embryo research until the 14th day after fertilisation.

**Main text:**

The article explores the most prominent arguments in favour of and against the extension of the 14-day limit for conducting research on human embryos. It situates these arguments within the history of the 14-day limit. I start by discussing the history of the 14-day limit in the United Kingdom and the reasons behind the decision to opt for a compromise between competing moral views. I then analyse the arguments that those who are generally in favour of embryo research put forward in support of extending the 14-day rule, namely (a) the argument of the beneficence of research and (b) the argument of technical feasibility (further explained in the article). I then show how these two arguments played a role in the recent approval of two novel techniques for the replacement of faulty mitochondrial DNA in the United Kingdom. Despite the popularity and widespread use of these arguments, I argue that they are ultimately problematic and should not be straightforwardly accepted (i.e. accepted without further scrutiny). I end by making a case for respecting value pluralism in the context of embryo research, and I present two reasons in favour of respecting value pluralism: the argument of public trust and the argument of democracy.

**Conclusion:**

I argue that 14-day limit for embryo research is not a valuable tool despite being a solution of compromise, but rather because of it. The importance of respecting value pluralism (and of respecting different views on embryo research) needs to be considered in any evaluation concerning a potential change to the 14-day rule.

## Background

In August 2016, in a letter in *Nature* and in an article published in *Nature Cell Biology*, two groups based in different research centres in the United Kingdom (Cambridge and London) and in the United States (The Rockefeller University, New York) presented the results of their experiments on in vitro human embryos. For the first time, the embryos were sustained in vitro for 12-13 days after fertilisation [1, 2]. Prior to this, scientists were only able to sustain embryos in vitro for about seven days [3].

Many members of the scientific and bioethics communities reacted enthusiastically to these advances, due to the novelty of the results and to the potential benefits that they could bring about [3–5]. Research involving human embryos allows us to increase our understanding of the first stages of embryo development and it is considered instrumental to shedding light on the causes of early miscarriages, of problems related to infertility and of birth defects [6]. In addition to this, embryo research has been instrumental to the development of human embryonic stem cells, cells derived from embryos have proved to be clinically useful to cure certain degenerative diseases [6–8]. Sustaining embryos in vitro for a longer period of time could allow an even greater understanding of the causes of embryo defects and early miscarriages, and it could prove especially clinically beneficial for women who have experienced multiple early pregnancy losses. Due to the current benefits of embryo research and to the potential future benefits of it, the positive reactions to these experiments went hand in hand with a call for revising and extending the so-called 14-day rule. This rule allows research involving human embryos up until the 14th day after fertilisation, a statutory binding limit in over a dozen countries [3, 9].

This article explores the arguments for and against extending the 14-day limit for research on human embryos. In the following section, I briefly present the history of how the 14-day rule came about in the United Kingdom and the reasons behind the decision to opt for a solution of compromise. In section 3, I discuss the arguments that those who are generally in favour of embryo research put forward in support of extending the 14-day rule, namely the argument of the beneficence of research and the argument of technical feasibility (further explained below). I show how these two arguments played a role in the process that led to the approval of mitochondrial replacement techniques in the United Kingdom. In section 4, I discuss why I find these arguments wanting. In the last section (5), I present two arguments in favour of compromise, namely the argument of trust and the argument of respect for value pluralism. I conclude that the importance of respecting value pluralism needs to be taken into account in any evaluation concerning a potential change of the 14-day rule.

## The 14-day limit and the Warnock report

The publication of the aforementioned two articles in *Nature* and *Nature Cell Biology* triggered a resurgence of the debate on embryo research and on the 14-day limit to carry out research on in-vitro human embryos. The 14-day limit came about in the United Kingdom at the beginning of the 1980s. Its birth is closely linked to another, non-metaphorical, British birth: the first test-tube baby (i.e. a baby conceived via in-vitro fertilisation), Louise Brown, was born in the United Kingdom in 1978. As noted by historian Duncan Wilson, after the initial excitement surrounding Louise Brown’s birth, public attitudes towards IVF shifted from an initially more favourable stance to a more critical view of the practice [10–12]. These predominantly negative attitudes, and the necessity to decide upon the fate of embryos ‘left over’ after IVF procedures,^1^ contributed to calls for a tighter oversight of the practice. They also underscored the importance of deciding whether it was permissible to use these spare embryos for research [10–12].

At that time, embryo research was the most debated matter concerning the ethics of IVF [13–15]. Two conflicting positions dominated the public debate: on the one hand, those of whom were outright against embryo research. On the other, those of whom were in favour of doing research on embryos up until it was technically feasible. The first group appealed to the need to respect human life from its very beginning and argued that life starts in the moment of fertilisation (i.e. when sperm cells fertilise oocytes) and must be protected. Interestingly, not all the opponents of embryo research holding the view that embryos are persons were arguing from a religious standpoint [15]. Some of those arguing against embryo research in principle referred to the potentiality of the embryos to become fully developed persons and concluded that human life, no matter at what stage of development, should be granted full protection, and that embryos should not be used for research [16–18]. The opposing view, held by those in favour of legalising embryo research, found support from those appealing to the potential benefits of such research, and from those who granted inexistent or low moral status to the embryos. This group also referred to the potentiality of embryos to become fully developed persons, but concluded that potential persons (i.e. embryos) were different from actual persons and that this was a sufficient reason to allow research on human embryos [13]. Unsurprisingly, according to them, the potential benefits of such research, for instance an increased understanding of early human development, better IVF procedures and treating infertility and pregnancy losses outweighed the costs of embryo research [13].

There are some differences between the 1980s debate on embryo research and today’s newly emerged debate. Perhaps, the main difference is that, whereas previously research beyond the 14-day mark was scientifically untenable, it has recently become technically possible. When the limit was decided upon, scientists were not able to keep the embryos alive in vitro for longer than the limit allowed. The experiments reported in the two recent articles prove that scientists are now able to keep embryos alive for up to 12-13 days and possibly longer. In addition, IVF as an assisted reproductive technique has significantly improved and many of the technical advances in this technique are owed to embryo research. It is in this sense that, while the 1980s debate focused on the question of whether embryo research should be allowed, the current debate occurs against the backdrop of the advances that allow embryo research to be made possible. Moreover, while in the past it was not possible to preserve the viability of the embryos employed for research, today there are technical solutions that allow scientists to obtain embryonic stem cells for research that do not result in the destruction of the embryo (e.g. embryo biopsy^2^). Lastly, whilst previous research was carried out on early human embryos only, today, and potentially increasingly in the future, embryo research could be done on artificial entities that bear sufficient resemblance to embryos to be suitable for such research. To name a few methods, these entities would be created through, for instance, altered nuclear transfer (ANT) or parthenogenesis of oocytes [6, 7, 19].^3^

### Conflicting moral views on embryo research

Today’s discourses on the moral status of human embryos are not so different from the discourses that, in the 1980s, resulted in the establishment of the *IVF Inquiry*, a committee appointed to produce an advisory report on the moral, legal and social issues raised by IVF, embryo research and other practices. Oxbridge philosopher Mary Warnock was appointed its chair. As I show in the next sections, the procedural work of the committee, the views of the chair, and the way the recommendations on how to proceed about embryo research were drafted represent an important precedent for the current debate on embryo research.

The members of the committee, including Warnock herself, were aware of the conflicting moral views on embryo research, and of the difficulty of reconciling them and establishing which one should prevail [20–22]. In addition to this, they tried to review as many different points of view as possible: the committee considered evidence from experts working in the field of human reproduction (around 300 individuals and organisations) as well as from the public (695 letters and submissions). Although the evidence collected in this way was never published^4^ and although it was never made transparent how this evidence influenced the final recommendations, it is presumed that the committee considered all the submitted evidence and took into account the different views that it reflected [15].

Legitimating embryo research would have likely caused uproar from those who accorded full moral status to human embryos. At the same time, an outright ban on embryo research was perceived as problematic for two reasons: due to a concern for the loss of potential benefits of embryo research, and due to the perceived need to allow IVF to go forward only if backed up by studies on the development of early human embryos. A solution to this impasse was to find a compromise between these two positions: this is how the idea to introduce a cut-off point until which research would be permissible came about. Introducing a cut-off was a solution of compromise, as it would have enabled embryo research, but only until a certain stage of development. Different possible limits were examined, including the 5th day (i.e. beginning of implantation in utero) and the 11th day (i.e. the end of implantation) after fertilisation.

It was developmental biologist Anne McLaren, a member of the committee, who proposed using a peculiar biological event in the embryo development to mark the end of the permitted period of research [11]. McLaren suggested limiting research to the 14th day of development because this moment signals the emergence of the primitive streak in the human embryo, a precursor of the brain and the spinal cord. At the same time, the emergence of this streak marks the beginning of gastrulation, a process whereby the embryonic inner cell mass starts to differentiate into three layers (endoderm, mesoderm, and ectoderm). This process also corresponds to the last point in which the embryo could cleave into twins (i.e. twinning) or in which two embryos could merge into one (e.g. tetragametic chimerism). McLaren argued that: “If I had to point to a stage and say ‘This is when I began being me”, I would think it would have to be here” [23]. In order to endorse the 14-day limit and the decision to allow research up until this stage of embryo development, the term ‘pre-embryo’ was coined. It designated the embryo before the emergence of the primitive streak, and it marked a distinction from the ‘unborn child’ (i.e. the embryo after the 14-day) [12, 23]. It was therefore a term with ethical and political significance, a term that designated the boundary between acceptable and non-acceptable research.

Eventually, in 1990, the recommendations of the IVF-Inquiry comprised in the Warnock Report [21] were enshrined into law, in what became the Human Embryology Act [22].

### How the 14-day limit came about: Compromise and its critics

Introducing a cut-off date –in this case the 14-day limit – represented an instance of favouring compromise between competing moral views, beliefs and values over questions of rightness and wrongness [10, 15, 24]. Questions regarding whether or not the embryo has moral status, what moral status stands for and entails, and questions regarding the core features of personhood and the beginning of human life were overridden by other considerations. These considerations included the moment from which the embryo should be granted legal protection, what kind of society can be praised and in what kind of society people can live with clear conscience [12, 14]. The decision to shift the focus from ontological questions concerning rightness and wrongness to more practical questions is linked to a conception of morality whose role is to address moral matters arising in the context of public policy. The IVF-Inquiry was not created to produce perfect philosophical reasoning and give a lesson in moral expertise, but rather to facilitate a process whereby scientists’ work would become more “socially palatable” and whereby workable regulations would be delivered [12, 25].

The committee favoured a moral relativistic approach to embryo research and to the conflicting positions present in the debate. Instead of trying to establish which position was the most accurate one and what view came closest to an absolute moral truth, the committee worked under the assumption that the views of those for and against embryo research deserved to be equally respected and taken into consideration. Thus, the view of those who believed that the embryos are to be treated as if they were persons (and hence, they deserve full moral status) and research on them should be banned, and the view of those who believed that embryos are not more than a cluster of cells (no moral status at all) and research on them should go forward were equally taken into account. In this sense, the committee followed the assumption that the truth and standing of moral judgments is not universal, but relative to the social, political and cultural context in which these moral judgements arise [26]. Warnock and her committee experienced first-hand the diversity of views both in her committee and in society at large. Their strategy was to exercise tolerance in matters of morality and moral disagreement, and to respect value pluralism [14, 27]. Warnock understood the role of her committee in these terms: starting from the acknowledgement of the different and competing moral positions, she tried to find the path of greater social consensus among them [10]. In addition to this, Warnock and her committee opted to take into account not only moral arguments based on scientific evidence and philosophical reasoning, but also moral feelings and beliefs [18]. In this sense, they followed Hume’s idea that feelings, and not pure calculating rationality, need to be considered in the assessment of ethical dilemmas and that morality is ‘more properly felt than reasoned’ [28, 29].

Perhaps unsurprisingly, given the existing disagreement on the matter, the committee recommendation to allow embryo research up until the 14th day was highly criticised. Three committee members were outright against embryo research and refused to endorse the final recommendations concerning this matter [15, 21]. Members of the conservative party, of the pro-life group LIFE and Christian scientists such as Ian Donald, publicly criticised the decision and lobbied against the report recommendation during the parliamentary debate on the matter [12, 15]. Generally, reactions from the more conservative side of the debate opposed this solution because it employed a sort of utilitarian calculus (i.e. the potential benefits of embryo research) instead of foregrounding considerations concerning how we ought to treat unborn persons.

Interestingly, both those against and in favour of conducting research on human embryos agreed on some of the reasons why the 14-day limit was at least problematic, if not completely wrong, namely arbitrariness and dodging the most fundamental question. Those that criticised the decision on the grounds of its arbitrariness argued that it was impossible to draw a morally and legally significant distinction between an embryo that was 13, 14 or 15 days old. However, supporters and critics of embryo research drew different conclusions from this impossibility to draw morally consistent lines: supporters argued that embryo research should have been allowed until it was technically feasible (i.e. until when the scientists could keep the embryo alive in vitro), while critics argued that embryo research should have been banned altogether. Another point of convergence between supporters and critics was the fact that Warnock and her committee did not address the questions of when life begins and when an embryo becomes a person. The decision to focus instead on the legal and moral rights of the embryo, without addressing the issue of what an embryo really is, was seen as extremely problematic by both sides. According to them, it was impossible to decide whether or not the human embryo deserved protection without establishing why it/she/he deserved protection, in other words whether or not the embryo was a person [13, 18].

In addition to these critiques, philosopher John Harris criticised Warnock and the committee for taking into account people’s feelings. Harris argued that not all feelings were moral feelings and not all of them deserved respect. According to him, moral feelings should be evaluated on their capacity to make the world a better place, to save lives and postpone deaths [13].

These reactions are important because they show that, back then as today, there is indeed a fundamental moral disagreement concerning early human life, how to treat human embryos and about the legitimate role of feelings and passions in public and regulatory discourses [30]. The reactions that followed the committee’s recommendations show the extent to which these views were in fact incompatible. However, it is important to note that those who criticised the decision on the grounds of arbitrariness and inconsistency in a certain sense missed the point of the role and function of the committee. The committee was put together in the first place in order to maintain public trust and be a reliable means for external oversight of scientific research. For this reason, the recommendations were meant to be a solution of compromise rather than a means to find the most consistent moral view.

In the next section, I briefly outline the reasons that advocates of embryo research currently put forward in favour of extending the limit, and show how these same reasons have played an important role in the debate on whether to introduce two new techniques into the clinic.

## The reasons in favour of extending the limit

Scientists (Robin Lovell-Badge and Azim Surani quoted in [4]) and ethicists [3, 5] reacted to the results reported on *Nature* and *Nature Cell Biology* by publicly calling for an extension of the 14-day limit and for revising the current regulation of embryo research. The argument that they used strikes familiar chords: embryo research is beneficial and now technically possible, therefore it should be allowed. The two publications in *Nature* and *Nature Cell Biology* [1, 2] partially changed the narrative of the debate on embryo research: whereas in the 1980s it was a matter of legalising such research, today the debate is about extending the 14-day limit for reasons grounded in beneficence and technical feasibility, and thus merely adjusting the regulatory framework of an already legalised practice. These reasons draw upon consequentialist premises and the principle of utility. They imply that being able to carry out potentially beneficial research and not doing so would be morally impermissible.^5^

According to the advocates of embryo research, the reasons in favour of extending the 14-day limit are stronger today than they were in the past. In 1984, these reasons relied on positive provisions of the potential benefits (i.e. the beneficence of research) and positive provisions of the future feasibility (i.e. technical feasibility). In the past, it was about faith in science and managing the uncertainties of potential future benefits of embryo research with certain regulations. Today, Harris, Lovell-Badge and Surani argued, it is about certainties concerning the benefits and certainties of technical feasibility: embryo research has proven to be both beneficial and feasible [4, 5].

The use of beneficence and feasibility in the debate on technical innovations recalls another debate where similar arguments have been advanced in response to scientific breakthroughs. Early in 2015, the United Kingdom became the first country in the world to allow two novel techniques that allow women with mitochondrial DNA diseases to have genetically related children with a decreased risk of developing mitochondrial diseases. Mutations in the mitochondrial DNA are the cause of many diseases including, for instance, mitochondrial myopathy, Leigh disease and diabetes mellitus, and they are normally inherited through the maternal line [31]. Up until the approval of these two techniques, prospective mothers needed to turn to oocytes donors, PGD or adoption in order to have children free from these genetically inherited mutations [32]. Although these techniques (maternal spindle transfer, MST, and pronuclear DNA transfer, PNT) have been depicted as involving the ‘replacement’ of the affected mitochondrial DNA of the oocyte of the prospective mother or of the fertilised oocyte with the mitochondrial DNA of a female donor, this description is inaccurate. What really happens is that the oocyte’s, or zygote’s, nucleus previously housed in a cell with deleterious mitochondria is rehoused in an enucleated cell with healthy mitochondria. The embryo that results from these techniques will have the genetic makeup of the prospective father, the mitochondrial DNA of a donor and the nuclear DNA of the prospective mother.

Despite the similarities between the arguments in favour of the extension of the 14-day limit and the arguments in favour of allowing mitochondrial replacement techniques (MRTs), it is important to note that there are differences between the current debate on extending the limit for embryo research and the recent debate on MRTs.^6^ These differences concern both the *content* of these debates (i.e. the specific arguments in favour and against and the object of the controversy) and their potential *outcomes* (i.e. extending an existing limit for embryo research instead of allowing two new techniques to be introduced into the clinic). With respect to the content, the arguments against MRTs focused on concerns regarding the implementation of newly developed techniques and the risks that their implementation may pose to future children. On the contrary, the arguments against the extension of the 14-day limit focused on basic research rather than clinical implementation. In particular, they pertain to the ethics of using intrinsically valuable beings such as human embryos for instrumental purposes. In addition, these debates differ in terms of what proponents and opponents wanted to achieve (i.e. in terms of outcome). The potential outcome of the debate on MRTs was to establish whether these new techniques were sound from a technical and moral point of view. On the contrary, the debate on embryo research is about setting a new limit for continuing existing research and for possibly gaining new insights into embryo development. These are just a few of the differences between the two debates and a detailed analysis of such differences is beyond the scope of this article. However, it is important to note that despite these differences, some similarities with respect to the argument in favour of MRTs and embryo research can be drawn. In particular, those in favour of MRTs and of extending the 14-day limit appealed to beneficence and technical feasibility arguments in both instances.

One of the most contested issues concerning the ethics of MRTs is whether these techniques would bring about changes to the human germline (i.e. changes in human oocytes, sperm cells or embryos that do not only appear in the children resulting from the procedure, but also in succeeding generations) [33]. Ethicists and scientists are divided over whether MRTs amount to germline modifications as changes introduced in the oocyte (in the case of MST) or in the zygote (in the case of PNT) concern the mitochondrial rather than the nuclear DNA [34]. In addition, as mitochondrial DNA is inherited from the maternal line, if only male embryos are transferred in utero, the modifications introduced with MRTs will not be present in the succeeding generations^7^ [35]. An assessment of these arguments is beyond the scope of this article,^8^ but what matters for the present analysis is that up until the approval of these techniques, modifications of the genetic makeup of sperm cells, eggs and embryos were only legally possible in-vitro and never for clinical purposes *in-vivo*. Modification of the human germline (i.e. gametes, and embryos) has traditionally been considered a line that should not be crossed. This line was recognised as morally relevant in 1978 with the publication of *Splicing Life*, a report of the US President’s Commission for the Study of Ethical Problems in Medicine and Biomedical and Behavioral Research appointed to regulate gene therapies, the reasons given were partly scientific (i.e. it was not technically feasible) and partly moral (i.e. it was seen as immoral to introduce changes that would have been inherited by future generations) [36, 37]. Modifying the human germline is seen as problematic because of the unforeseen effects on future generations, the risk of engaging in a form of new eugenics, the risk of sliding down a slippery slope to human enhancement, and other similar arguments [38–40]. These arguments were already put forward at the very early developments of gene therapy and rehearsed in recent debates on MRTs and gene editing [34]. However, both historically and more recently they have not remained unchallenged. Questions related to eugenics, enhancement and unforeseen effects on future generations have been widely discussed during the months prior to the approval of MRTs and they are still a matter of ethical inquiry, as shown by the increasing number of articles and reviews that address these issues [41–44]. In addition, the public consultation (2012) and the extensive reviews of the scientific methods of MRTs carried out by the HFEA (respectively in 2016, 2014, 2013, 2011), the work of the Nuffield Council^9^ [44] and the parliamentary debate on these techniques have considered such concerns. The 2015 approval of these techniques by the UK Parliament could be seen as a first instance of crossing an internationally recognised ethical and legal limit due to reasons of beneficence (i.e. children born with these techniques will be free from mitochondrial diseases), but also due to the technical feasibility of germline modifications (prior to the parliamentary vote on MRTs, these techniques were not considered safe enough to be introduced into the clinic) [45]. It is in this sense that the sum of the arguments in favour of extending the 14-day limit echoes, albeit only partially, those in favour of allowing MRTs. Mitochondrial replacement techniques represent an interesting case study and set an important precedent for the ethical assessment of technical innovation. In contrast with other instances of internationally recognised bans such as the ban on human cloning, the approval of MRTs shows that long-standing limits such as the ban on germline modifications can be redefined once scientific advances make it possible. The argument of beneficence to allow research on human embryos for longer than 14 days is the same as the one made in the 1980s. What has changed is that while before it was technically difficult to introduce changes in reproductive cells and embryos that would be inherited by future generations, and to keep the embryos alive in vitro for a longer time span, now both actions are theoretically possible. The question, therefore, is whether the potential benefits of embryo research and the feasibility of keeping the embryos alive for longer than ever before are sufficient reasons to extend the limit.

## There is more to beneficence and technical feasibility than meets the eye

In this section, I will show that technical feasibility and beneficence of research as reasons in favour of extending the limit of embryo research are not as fundamental as those who advocate this change in the law claim. Accordingly, I scrutinise the arguments in favour of the extension of the 14-day limit, while I leave unchallenged those presented by the advocates of a more restrictive regulatory framework for embryo research. The rationale behind this choice does not rest on my own view on embryo research, as I do not necessarily share the beliefs and values of those against this practice. However, it is often argued by proponents of technological changes that the burden of justifying one’s own claims rests solely on those who take a precautionary approach to technological progress [46–48]. Against this view, I propose that both those in favour and against embryo research ought to share the burden of justifying their moral views.

### Facts, values and rationality

Technical feasibility as a reason in favour of extending the limit relies (i.e. practice x is now technically feasible, so there are good reasons to change the rule) on the premise “practice x is technically feasible” to infer the conclusion “there are good reasons to change the rule”. However, appealing to the beneficence of research and to its technical feasibility is more problematic than those in favour of extending the limit for embryo research suggest it is. This line of arguing is problematic because it relies on what eighteenth-century philosopher David Hume considered an “inconceivable deduction” of what *ought* to be done from a set of *is*-premises [28]. Hume believed that it was logically fallacious to infer a normative judgment (ought-conclusion) from a set of factual claims (is-premises). Thus, following Hume, the normative conclusion “there are good reasons to change the 14-day rule” cannot be rightly inferred from the factual premise “embryos can now survive in vitro for longer than before” (i.e. technical feasibility of extending the time span for embryo research). This critique of inferring normative conclusions from factual claims is similar to the critique that philosopher George Edward Moore moved to moral naturalists (i.e. those who argue in favour of a link between moral philosophy and the natural sciences). Moore argued that anyone who infers that practice x is good from any preposition about the natural properties of x commits the “naturalistic fallacy” [49]. According to Moore, this fallacy shows how premises about some factual or natural features of practices do not support normative conclusions about these practices. Thus, anyone who supports an extension of the 14-day limit for embryo research on the basis of the technical feasibility of this research would commit the naturalistic fallacy. According to Moore, one of the main problems of moral naturalists was that they relied on purely factual premises concerning the natural features of certain practices to infer normative conclusions concerning these practices. To counter this tendency, Moore suggested instead that normative conclusions ought to be inferred from both factual and normative premises.

The argument of the beneficence of research (i.e. embryo research should be allowed for longer than 14 days due to the benefits of such research) is also more problematic than those in favour of extending the limit suggest it is. According to this argument, the 14-day limit should be extended because of the potential benefits of such research and because these benefits outweigh the costs of embryo research [5, 13, 50, 51]. This appeal to beneficence is common in bioethics and it is often used by those who take a utilitarian stance on the ethical assessment of scientific progress, technologies and practices [3, 46, 52–54]. Proponents of what I have called the argument of the beneficence of research rely on historical evidence to support their claim: they argue that since technological and scientific progress in medicine proved to be beneficial to humankind, it should be allowed to continue. Returning to embryo research, those who appeal to the beneficence of research to extend the 14-day limit ground their argument on the past benefits that embryo research brought about, and on the potential benefits that the extension of the limit could bring about [4, 5].

At first sight, it seems fairly obvious that if something is beneficial, even only potentially beneficial, it should be allowed. However, this approach is problematic for a number of reasons and scholars have criticised bioethicists, institutions and scientists for their often-hyped claims concerning the benefits of new technical possibilities [55–58]. Firstly, the argument of beneficence and its proponents rely on an optimistic view of scientific progress, research and technologies [55, 59, 60], a view that echoes the post-illuminist positivistic ideas of science and technology, and that often overemphasises the potential benefits of scientific research [56, 58, 59] and its understating as a progressive and linear endeavour [61, 62]. Secondly, the argument is problematic because it relies on a misleading estimation of costs and benefits. The benefits taken into consideration for the cost-benefit assessment are not the benefits of embryo research for the embryos, as embryo research does not benefit embryos. Instead, the benefits considered are those to society, to existing and future individuals. On the contrary, the costs taken into account for the cost-benefit assessment are not those to society, but to the embryos used for research. Those who emphasise benefits of embryo research over its costs do not grant moral status to the embryos, nor do they believe that embryos are capable of experiencing pain (i.e. being harmed). Hence, they do not really see any cost associated with embryo research, and they thus conclude that benefits outweigh these (inexistent) costs. The substantial disagreement over the moral status of the embryos and the criticism moved against research on human embryos show that embryo research is a controversial and not-settled issue [15, 63]. For this reason, the costs of extending the limit beyond the 14th day, and of embryo research more generally, might be higher than proponents of embryo research like to admit. Embryo research has a societal cost of offending certain moral feelings on the value of early human life, and not respecting certain strongly held convictions on how we ought to treat human embryos. Thus, individuals who hold such views may find themselves feeling alienated from or devalued by society [17, 18, 64]. Possibly, proponents of embryo research who argue from a utilitarian standpoint, and who rely on the argument of the beneficence of such research, are aware of the possibility of offending moral feelings and strongly held beliefs, but they still consider the benefits of embryo research greater than the costs of offending the people who hold these feelings.

One of the reasons why many proponents of embryo research do not grant moral worth to these feelings, and to the opponents’ arguments, is that they consider their views to be fundamentally flawed, irrational and not grounded in scientific evidence. Most advocates of embryo research thus dismiss the view that embryos are (future) persons and that embryo research would violate these future persons’ dignity on the grounds of the irrationality of such ontological claims. For to them, these claims are based on faith rather than reason and factual considerations. However, it is important to note that those in favour of embryo research who argue from supposedly rational positions do not live up to the very same standards of rationality that they require of their opponents. In this sense, dismissing questions related to human dignity and the moral status of the embryos on the basis of their irrationality and lack of scientific support, becomes problematic [65, 66]. Scientific evidence is often interpreted according to one’s own pre-existing moral convictions, so-called evidence-based claims are still influenced by these moral convictions and by the way bioethicists react and argue about new technical possibilities [56, 67, 68]. Thus, irrational beliefs are not an exclusive ownership of those arguing against embryo research: similar irrational beliefs play a role in assessments of embryo research put forward by those in favour of embryo research on the grounds that it can save future lives.^10^

### Slippery slope

The slippery slope argument offers a last reason of caution against embryo research [69–71]. The slippery slope argument entails that allowing practice x (in this instance, allowing embryo research or extending the limit for embryo research) would initiate a process leading to unethical practices w, y, z. The slippery slope argument against embryo research is approximately like this: embryo research should not be allowed/the limit should not be extended because allowing research on embryos in a very early stage of their development/extending the limit beyond day 14 will lead to the permissibility of research on foetuses and new-borns. The argument voices the concern that once we become accustomed to research on pre-embryos, we will extend the permission for research on embryos on a later stage of development; once we become accustomed to this too, then we will allow research on foetuses and babies. ‘Slippery slopers’ believe that morally problematic practices such as embryo research should not be allowed, or the limit should not be extended, because of the difficulties of drawing a line between practices currently considered less morally problematic, such as research on pre-embryos, and practices currently considered highly immoral, such as research on foetuses at a late stage of their development. These arguments are widely criticised in the philosophical arena for their lack of empirical evidence, and for not considering that government regulations can be used to prevent such scenarios from coming into being [72–74]. In spite of these critiques, they are still used in debates on technological advances, scientific research and policy making [68, 69, 71, 75]. The persistence of slippery slope arguments in academic works and policy making seems to suggest that attempts from philosophers to discredit this argument have been unsuccessful. The charge of starting a slippery slope towards inadmissible practices is still a powerful one [63, 68]. An analysis of the theoretical fallacies and merits of this argument is beyond the scope of the paper, as is a final assessment of its validity. However, it is important to note that extending the limit beyond the 14th day of development will provide support to those who rely on the slippery slope argument to oppose embryo research. This might have non-negligible social consequences. For example, extension of the limit for embryo research would show that what is feared by ‘slippery slopers’ (i.e. that once a practice becomes legal it is difficult to prevent the permission of its future developments) can eventually become a reality. Even if the limit was extended only for a few days, ‘slippery slopers’ might take this extension as a sign that their fears are well grounded, contrary to what their critics argue.

## Is compromise the best way forward?

Let me take stock of what I have said thus far. In the previous section, I have shown how the arguments of beneficence and technical feasibility in favour of embryo research and of extending the 14-day limit are less straightforward than their proponents seem to suggest. I have also suggested, using the slippery slope argument as an example, that extending the limit for embryo research might undermine public trust in scientists, regulators and overseeing bodies. In order to show the importance of compromise and the value of respecting pluralism in the context of embryo research, I will not juxtapose the arguments of the beneficence of research and of technical feasibility with arguments pertaining to the sanctity of human life and human dignity. These arguments arise in the context of fundamental disagreements concerning the beginning of human life, the value of personhood, and concerning what respect human dignity ought to entail. They are portrayed as factual questions by both advocates and critics of research (i.e. research beyond the 14-day should not be allowed/should be allowed because human embryos *are/are not persons* and doing research on them would/would not violate their dignity); however, they are not merely a matter of fact, but they are informed and shaped by values, feelings and beliefs. Regardless of one’s opinion regarding the values and beliefs of those defending the sanctity of life view, the burden of justifying one’s claim should rests both on those defending this view and on those advocating technological progress, contrary to what seems to be normally believed [48].

What I intend to argue in this last section is that even if the question of the moral status of the embryos cannot be easily settled, there are two arguments in favour of reaching a compromise and respecting value pluralism in the context of embryo research: the argument of trust and the argument of respect. I argue that the argument of trust in favour of compromise, albeit being sound and widely used, could, in certain instances, assume instrumental and paternalistic forms. I then argue that in the context of embryo research and more generally in the governance of scientific and technical breakthroughs it would be helpful to employ what I call the argument of respect.

### The argument of trust and the argument of respect

The first argument in favour of reaching a compromise that, other things being equal, respects value pluralism is what I define as “the argument of trust”. It is structured as follows:

- Scientific research is important because it improves people’s lives and it should be allowed to carry on
- Public trust is necessary to carry on scientific research
- Therefore, public trust in scientific research ought to be preserved

Given competing views concerning the moral status of the embryo, this argument provides a reason in favour of finding a solution of compromise that accommodates as much as possible these views and avoids the risk of overriding those of one camp with those of the other. The argument of trust relies on premise a) to show that people’s lives are improved by scientific research [76]. It relies on premise b) to show that public trust is a necessary condition for scientific research to be carried on [77, 78]. Trust is needed to ensure public acceptance of concrete applications of research; to preserve public confidence in policies informed by scientific research; and to allow the investment of public resources in scientific research [77, 78]. In the context of embryo research, the argument shows that, given the potential benefits of embryo research (premise a), and given the importance of public trust to carry on this type of research (premise b); there are good reasons to preserve public trust (conclusion c). Following this argument, it is possible to draw two conclusions: on the one hand, if the extension of the 14-day limit for embryo research is strongly opposed by the public,^11^ then there are good reasons not to extend the limit. On the other, if opposing views coexist in the public understanding of embryo research, then there are good reasons to find a solution that strikes a compromise between these views.

The 14-day limit was a solution of compromise between conflicting moral views designed to maintain public trust whilst allowing research to go forward [12, 24, 79]. Today, there are two questions that need to be addressed, an empirical and a normative-theoretical question. The empirical question is whether the public (or at least a vast majority of it) is against the extension of the 14-day limit for embryo research. The normative-theoretical question is whether public opinion should influence the decision to change or retain the current 14-day rule, and if so, to what extent. An implication of taking into account the empirical question is that, if the public view of embryo research has become more favourable, then there is at least one good reason in favour of revisiting the 14-day rule.^12^ In January 2017, a YouGov poll commissioned by the BBC in the United Kingdom, asked respondents’ views on an extension of the limit up to the 28th day. Interestingly, 48% of the 1740 respondents said that they would be in favour of extending the limit, while 19% wanted to keep the current limit. In addition to these respondents, 10% maintained that they would want embryo research to be banned altogether, while 23% did not express any of the aforementioned preferences [80]. In addition to the empirical question regarding public attitudes towards the extension of the 14-day limit, one may wonder how such attitudes would be towards therapies and scientific results obtained thanks to research on embryos beyond this limit in countries that may extend it. Currently, the 14-day limit is either enshrined in the laws (for instance in the United Kingdom, Canada and Spain) or specified in the scientific guidelines (for instance in Singapore, China and in the United States) of many countries. However, these regulatory frameworks may change in the future. Hence, if this becomes the case, it would be interesting to investigate public attitudes towards those therapies and other advances of basic research that are made possible by research in countries that allow embryo research beyond day 14.^13^

I will not provide an answer to these empirical questions here, if only because of the dearth of empirical data on public attitudes towards the extension of the limit, and embryo research more generally. Regarding, instead, the normative-theoretical question (i.e. whether public opinion should influence the decision to change or retain the current 14-day rule) the argument of trust would indicate that the answer is yes: public opposition to extending the 14-day rule should prevent its extension, while public agreement to a proposed change (i.e. the 28-day limit or other future proposals) should facilitate its extension. The risk of proceeding regardless of public attitudes towards an extension of the limit is that policies derived by embryo research will not be backed up by public consensus and applications of embryo research (e.g. therapies developed thanks to the knowledge yield by embryo research) not accepted. If the importance of maintaining public trust in scientific research (premise b) is motivated by these considerations, *then* it seems that public trust is only valued for instrumental and extrinsic reasons. In other words, this understanding of the importance of maintaining public trust in scientific research does not value public trust for its own sake, but only for its role in allowing research to go forward. What is problematic of this approach to public trust is that it offers a consequentialist reason in favour of respecting value pluralism, a reason that pertains to the better tangible outcomes of respecting value pluralism over other strategies of governance. In addition to this, when the instrumental justification of maintaining public trust is associated with a representation of the public as ill-informed and with little or no understanding of the potential benefits of research, it could be motivated by paternalistic considerations. Scientists and ethicists may risk misinterpreting public concerns and views over embryo research as the result of a lack of expertise or evidence-based information rather than a matter of legitimate and genuine disagreement over values [81, 82].

The second premise of the argument of trust, however, could be also motivated by a concern for a deliberative conception of democracy. This conception of democratic governance requires to both citizens and their representatives to provide public justifications of their views and to engage in deliberative processes. Public trust becomes then fundamental to allow these deliberative processes to take place and to foster better strategies for policy-making [82, 83]. These deliberative processes of mutual exchange between experts and the public, together with a commitment to respecting conflicting moral views (i.e. respect for value pluralism) provide a reason in favour of finding a solution of compromise that, given competing views concerning the moral status of the embryo, respect this plurality of views and values regarding embryo research. These considerations concerning the importance of maintaining public trust echo other considerations employed to defend democracy as a political system and as a valuable form of governance. These include, for instance, equality: given the existence of conflicting views, values and beliefs, a good reason to respect them is that people or groups holding these different views will be respected by being granted an equal say on matters of common concern [84, 85]. Mertens and Pennings [8] have argued in favour of the benefit of compromise in the context of different policies regulating embryonic stem cell research and have concluded that there is a moral obligation to respect conflicting moral views [8]. Similarly, Devolder argued that in spite of the epistemic costs of compromise, middle-ground positions could still be defended in the context of policy-making [6]. What I suggest here is that the commitment to a democratic decision-making process entails a fundamental respect for value pluralism [86]. In Warnock’s and the IVF-Inquiry’s time, this respect for value pluralism translated into a deliberation resulting in the 14-day rule. Today it translates into favouring an assessment of the rule and of the potential reasons to change it that once again takes into account the conflicting moral views held in society; an assessment that cannot rest on the argument of the benefice of research and of scientific feasibility alone.

## Conclusions

In this article, I have argued that the 14-day limit for embryo research is not valuable *in spite of* being a solution of compromise, but rather *because of* it. The idea of a democratic society is that even those who do not accord intrinsic value to the human embryo should respect value pluralism and accord moral worth to opposing views. For this reason, any proposal to change the 14-day rule needs careful evaluation of the scientific feasibility and effective benefits of embryo research; it needs an extensive inquiry into public attitudes concerning embryos; and it needs a deliberative process that takes these elements into account. It does not need positions that consider only the beneficence of research and its technical feasibility. This would be undemocratic and potentially a move not backed up by a rigorous assessment of the science behind embryo research. Warnock and the other members of the IVF-Inquiry, albeit possibly guided by utilitarian-inspired views, opted for valuing a solution of compromise over other solutions [87, 88] They did so behind closed doors. In this sense, the recent experiments published in *Nature* and *Nature Cell Biology* and the newly sparked debate on embryo research represent a valuable opportunity to begin a truly deliberative and democratic debate on this issue [82, 86]. All in all, greater technical potential translates into greater responsibilities and need for deliberation.

## Footnotes

1 These embryos are not implanted in utero but frozen for further implantation. When a successful pregnancy is established, it had to be decided what do with these supernumerary frozen embryos.

2 For a detailed analysis of this alternative and of its limits, see the work of Katrien Devolder [6].

3 It must be noted that these two alternatives have been criticised for a number of reasons. For instance, it is unclear whether parthenotes are significantly different from human embryos and whether ANT really escapes the ethical challenges of embryo research and whether it is a scientifically realistic alternative [6].

4 The submissions from the experts can be found at the House of Commons Library, but they have never been published.

5 I commented elsewhere that this line of argument is problematic [61].

6 I am grateful to one of the reviewers for raising this point.

7 In the United Kingdom, the law regulating MRTs allows both female and male embryos to be transferred in utero. This is different from the American approach to the clinical implementation of these novel techniques: the National Academies for Science, Engineering and Medicine (NASEM) Report recommended that only male embryos should be implanted in utero [35, 89].

8 For insightful analyses of the MRTs debate and of the ethics of these techniques, see [41–43, 90, 91].

9 The Nuffield Council on Bioethics is an UK-based independent institution that examines ethical issues arising in the field of biotechnology and biomedicine.

10 For a detailed discussion of such position in another context (i.e. the debate on human enhancement), see [48].

11 It must be noted that the idea that ‘the public’ is against scientific developments and breakthroughs is criticised for being artificially constructed (see for instance [58]).

12 Other good reasons include technical feasibility, public utility and so forth.

13 A case in point is Germany, which allows research on embryonic stem cells that are produced abroad (i.e. in countries with less restrictive legislations) before January 2002 (when the German Stem Cell Law was issued), but does not allow to derivation of stem cells from supernumerary embryos [6].
